# Perceptions of Older Adults with Hematological Cancer on Diet and Exercise Behavior and Its Role in Navigating Daily Tasks

**DOI:** 10.3390/ijerph192215044

**Published:** 2022-11-15

**Authors:** Alana Colton, Monica A. Smith, Suzanne Broadbent, Karina T. Rune, Hattie H. Wright

**Affiliations:** 1 School of Health and Behavioural Sciences, University of the Sunshine Coast, Sunshine Coast, QLD 4556, Australia; 2 Australian Centre for Pacific Islands Research, University of the Sunshine Coast, Sunshine Coast, QLD 4556, Australia; 3 Sunshine Coast Health Institute, Birtinya, QLD 4575, Australia

**Keywords:** self-management, physical activity, nutrition, health behavior, survivorship

## Abstract

Diet and exercise are associated with the maintenance of physical function, independence and better health-related quality of life in cancer survivors. Adherence to healthy diet and exercise guidelines, however, remains low. The aim of this study was to explore the perceptions of hematological cancer survivors (HCS, ≥50 years) on the role of diet and exercise in navigating daily tasks using a qualitative descriptive research method. Eligible HCS completed an online survey gathering demographic information including physical functioning, exercise frequency, malnutrition and frailty risk. Following a semi-structured telephone interview, thematic analysis was used. Nine HCS (67 ± 2 years) were included in the final analysis, with 55.5% sufficiently active, three at risk of malnutrition and five of frailty. Three primary themes reflected the survivors’ perceptions: (1) beliefs about the impact of diet and exercise on physical and mental wellbeing, (2) the ability to overcome barriers to adhere to healthy diet and exercise behavior, and (3) diet and exercise empowered and gave hope. Participants had a more nuanced understanding of the role of exercise in physical function but lacked insight into the role of a healthy diet. Knowledge, support and instruction were key enablers of diet and exercise behavior, with community connection a unique enabler identified in this group.

## 1. Introduction

With significant gains achieved in antineoplastic treatment and improved survival of people suffering from hematological cancer, there is an increased focus on the quality of life of these cancer survivors. Up to 50% of hematological cancer survivors live beyond 10 years post-diagnosis and experience the long-term effects of the disease and cancer treatment, which impact their quality of life [1,2]. Both diet and exercise behavior may positively influence the quality of life of cancer survivors [3,4,5]. In addition, the implementation of healthy lifestyle behaviors, such as diet and exercise, may reduce the risk of recurrence [6], support treatment tolerance and survival in those on long-term maintenance treatment [4,7], as well as aiding in the management of comorbidities [4].

Reduced physical function is often reported by hematological cancer patients and is associated with poor overall quality of life. A recent systematic review and meta-analysis showed that low muscle mass was associated with poorer physical functioning domain and global health-related quality of life (HRQOL) scores, as well as a longitudinal association between changes in skeletal muscle and several HRQOL domains in adults with cancer [8]. Loss of muscle mass and strength in those with hematological cancer seem to be attributed to the interplay among the cytotoxic effects of chemotherapy [9,10], the treatment’s side-effects and the disease itself [11,12,13], resulting in reduced oral intake and low energy. The latter negatively impacts engagement in daily tasks and being physically active, which results in a vicious cycle of loss of muscle mass and strength [7,14], reduced physical function and poor HRQOL [15]. In addition, older cancer survivors are at a particular risk of losses in muscle mass and strength as a result of the aging process and the presence of comorbidities [16,17].

Physical activity guidelines for cancer survivors include 150 to 300 min of moderate to vigorous aerobic exercise and two strength training sessions a week [18]. Meeting these guidelines have benefits for cardiovascular and physical function and can improve the quality of life of cancer survivors [18]. In fact, regular exercise at a moderate intensity has been shown to significantly reduce fatigue and improve the quality of life in people with hematological cancer [19,20,21], particularly if sustained over time [22]. Despite the known benefits of regular exercise, adherence to physical activity guidelines amongst hematological cancer survivors (HCS) is mostly low [20,21,23]. A systematic scoping review on the factors influencing participation in physical activity by cancer survivors identified physiological, psychosocial and cultural, as well as economic and environmental factors as the main contributors to adherence to physical activity guidelines [24]. The majority of studies included in the review were from solid tumor streams or mixed cancer types, highlighting a paucity in the literature on the factors contributing to adherence to physical activity guidelines in HCS. A cross-sectional study amongst multiple myeloma (*n* = 229, 53.1% male) survivors found low overall adherence to aerobic exercise guidelines, with fatigue, injuries, pain, other health conditions and age-related decline reported to be the main barriers, with pre-diagnosis physical activity levels being the main predictor of adherence post-treatment [23]. Similarly, Shallwani and co-workers identified cancer- and treatment-related factors such as barriers to physical activity in multiple myeloma patients undergoing chemotherapy [20]. Factors associated with improved adherence to both aerobic and resistance training guidelines included younger age, fewer comorbidities and a higher level of education in a Canadian cohort of HCS [25]. Insight into the perspectives of HCS on their physical activity behavior is required to support survivors to improve their self-management of a regular exercise routine throughout their cancer journey.

As mentioned earlier, healthy eating principles are promoted post-treatment to manage comorbidities, improve the quality of life and reduce the risk of cancer recurrence [26]. In addition, healthy dietary patterns are associated with musculoskeletal health, which is important for physical function, reduced fracture risk and preservation of muscle mass in older adults and people with chronic disease [27,28,29]. Cancer survivors are aware of the importance of nutrition during the cancer journey [30]; however, long-term adherence can be low [31]. Contributing factors to dietary adherence include modifiable (i.e., being organized, knowledge, confidence, self-motivation, access to healthcare professionals) [31,32] and non-modifiable (i.e., age, time since diagnosis) factors [31,33,34]. Insights into the factors associated with adherence to healthy eating principles in older HCS are lacking.

The majority of studies investigating the barriers and enablers to healthy lifestyle behaviors to date have been in survivors with solid tumors, particularly breast, colorectal or prostate cancer [24]. Due to the distinct difference in disease outcomes and the treatment pathway of solid vs. non-solid tumors, more research is warranted into the factors affecting adherence to diet and exercise behavior in HCS. In addition, there is a limited understanding of the unique factors that may affect adherence to the diet and exercise behavior of older cancer survivors [35]. Furthermore, the body of evidence on diet and exercise adherence in studies including HCS is mostly quantitative in nature [23], which does not allow an in-depth insight into all the potential contributing factors. Finally, it is not clear whether cancer survivors receiving long-term maintenance treatment are facing unique challenges compared with those post-treatment. Research is warranted on the perspectives of older HCS on the importance of diet and exercise for living with and beyond a cancer diagnosis. Therefore, this study aimed to explore the perceptions of older adult hematological cancer survivors on diet and exercise behavior and their role in navigating daily tasks whilst undergoing treatment.

## 2. Materials and Methods

A qualitative descriptive research method was used to gain a deeper insight into the perceptions of older adults living with hematological cancer of their diet and exercise behavior. Qualitative description was selected, as it sought information directly from those with a shared experience of the phenomena and allows the opportunity for new or unexpected understandings to arise [36]. The findings were reported according to the consolidated criteria for reporting qualitative research (COREQ) [37]. This study sought to answer the following research questions. (i) What are the perceptions of older adults with hematological cancer undergoing treatment on the role of diet and exercise in their ability to engage with daily tasks? (ii) What factors affect the diet and exercise behavior of older adults with hematological cancer undergoing treatment?

### 2.1. Participants and Sampling

Participants aged 50 years and older who had been diagnosed with any type of hematological cancer and currently receiving antineoplastic treatment (including stem cell transplant recipients), and who were proficient in English or had access to an interpreter were eligible for inclusion in this study. Exclusion criteria included receiving end of life care or currently not receiving antineoplastic treatment.

### 2.2. Recruitment and Consent

Recruitment occurred through flyers at local medical centers, pharmacies and notice boards, as well as recruitment letters through Cancer Alliance Queensland. Informed consent was obtained from all participants prior to data collection. Ethical approval was gained from the University of the Sunshine Coast, Human Research Ethics Committee (S201464).

### 2.3. Data Collection and Procedure

Eligible participants first completed an online survey to gather demographic information, as well as their exercise frequency, understanding of physical activity guidelines [38], physical function, frailty and malnutrition risk to gain a deeper insight into their diet and exercise behavior and potential barriers to these behaviors. Physical function was assessed with the physical functioning domain of the 36-item short form survey (SF-36) [39]. The Godin leisure time exercise questionnaire measured exercise frequency [40], the Program of Research to Integrate Services for the Maintenance of Autonomy-7 (PRISMA-7) assessed frailty risk [41], and malnutrition risk was identified through the malnutrition screening tool (MST) [42].

Upon completion of the online survey, participants took part in a semi-structured telephone interview consisting of a series of open-ended questions exploring perceptions of the role of exercise and nutrition on physical function and factors influencing these behaviors. The questions were refined after the first two interviews, and minor amendments were made. For example, one was rephrased from “What is the role of your diet on your ability to do your daily activities?” to “How do you think what you eat helps you to do your daily activities?” Pilot data were not included in the final data analysis. Interviews were conducted by the second author between April and July 2021, and lasted between 15 and 45 min. Interviews were audio-recorded and transcribed verbatim by AC.

### 2.4. Data Analysis

Quantitative data were analyzed with IBM SPSS statistical software package (version 27). Continuous data are presented as the means ± standard deviation for parametric variables, and the medians and interquartile ranges for non-parametric variables. Categorical data are presented as frequencies and percentages of the total sample.

Thematic analysis was conducted according to the six-phase process of Braun and Clarke [43], namely familiarization with the data, initial coding, searching for themes, reviewing themes, defining and naming themes, and writing up the findings. Directly after each interview, the interviewer (MS) reflected on and recorded the key points that emerged from the interview. AC immersed themselves in the data through listening to the interviews several times and then completed the verbatim transcription. AC and HW completed the initial coding of two transcripts independently, compared and contrasted the initial codes and reached a consensus on the codes. Coding was recorded using the QSR Nvivo Software (Version 20, QSR International Pty Ltd., Australia) program. AC coded the remaining transcripts, and identified and defined the initial themes. Weekly meetings were held between AC and HW and AC kept a reflexive diary during the analysis to ensure that the analysis was based on the participants’ words and not the researcher’s assumptions. AC (a dietitian and research student) and HW (a dietitian and cancer researcher with expertise in qualitative analysis) identified and defined the final themes through an iterative process, which was reviewed by MS (the interviewer, a research assistant with training in nutrition sciences), KR (a researcher in psycho-oncology with expertise in qualitative analysis) and SB (a cancer researcher and exercise physiologist) to triangulate the analysis process [44]. The reflexive notes taken by MS after the interviews were considered during finalization of the themes and subthemes, and all researchers agreed on final themes.

## 3. Results

In total, 13 participants were recruited into this study. Four participants were excluded from the analyses, one did not complete the online survey and three did not meet the inclusion criteria (i.e., younger than 50 years and not currently receiving treatment), which resulted in a final sample of nine participants.

The participants characteristics are provided in Table 1. Most participants (*n* = 6) reported not having received dietetic services. Three participants were identified as being at risk of malnutrition and five were identified as being at risk of frailty. Two participants reported a significant impact on food intake due to treatment over the past four weeks, three reported moderate and four no impact. Five participants were unsure of the current physical activity guidelines, two incorrectly identified the guidelines and two correctly identified the guidelines.

### 3.1. The Role of Diet and Exercise in the Ability to Engage in Daily Tasks

Individual participant characteristics relating to gender, age, time since diagnosis, cancer diagnosis, physical activity level, physical functioning, frailty and malnutrition risk are summarized in Table 2.

Three main themes emerged from the analysis of participants’ perceptions of the role of diet and exercise on their ability to engage in daily tasks namely: (1) beliefs on the impact of diet and exercise on physical and mental wellbeing, (2) the ability to overcome barriers to adhere to healthy diet and exercise behavior, and (3) diet and exercise empowered and gave hope.

#### 3.1.1. Theme 1. Beliefs on the Impact of Diet and Exercise on Physical and Mental Wellbeing

This theme illustrates the participants’ beliefs regarding the importance of diet and exercise in navigating their daily life within the context of their cancer diagnosis. For most participants, diet and exercise were identified as important contributors to both physical and mental health, which enabled engagement in daily tasks, for example, “I think healthy diet and exercise are paramount to emotional wellbeing; there’s a connection there”, (Craig) and “I’m an advocate for exercise of any description to help you with your daily routine”. (Louise) and “I think it is just the... good food, that I am able to do what I want. [It] might just be some errands... I am able to do the things I want because of my diet”, (David).

An appreciation for the importance of following a healthy diet was shared by most participants, and strong ideas on what constitutes a healthy diet were voiced. Healthy food choices were associated with energy to complete daily tasks and exercise, feeling good, improved immune function, supporting overall health, tolerating treatment and helping with recovery. As described by Craig:

*I think healthy food creates a healthy body. What we eat today walks and talks tomorrow. That’s my attitude. If I’m gonna look after myself, and I want tomorrow to be good, I’ll eat well today.*

Furthermore, one participant who had been a bodybuilder when they were younger was aware of the importance of food in maintaining muscle mass and strength.

There was agreement that unhealthy food was associated with poor health and leaving one without sufficient energy to engage in daily tasks. As Samuel explained:

*If you eat* [takeaway meal] *every day, you’re not gonna get anywhere … it is not the type of food that will substantiate a decent level of vitamins and minerals and everything that you need for your body. … the right food gives you the power for your muscles to work properly and the amount of antioxidants and things like that. So, it’s still a matter of building muscle, but the right food is basically the building blocks of your muscle.*

Regular exercise was valued as an important facilitator to engage in daily tasks and remain active during the day. Furthermore, regular exercise was associated with psychological wellbeing, for example: “I think getting out and about doing some regular exercise helps with the mental aspect” (Kevin). In addition, participants reported that exercise gives energy, increases both fitness and cardiovascular health, helps to improve treatment-related symptoms and sleep quality, and gives confidence to do daily tasks. As described by Barbara:

*I find that it* [exercise] *makes me a little bit more alert, ‘cause one of the things that I have found is that my speech has been affected and I find doing the exercise must do something to the brain to help me to put sentences and that together’. Going through chemo treatment, you lose a lot of balance and exercise helps you get that balance back.*

A lack of awareness of the benefits of exercise was raised by one participant who reported to never have taken part in any formal exercise both prior to and after their cancer diagnosis.

#### 3.1.2. Theme 2. The Ability to Overcome Barriers to Adhere to a Healthy Diet and Exercise Behavior

This theme describes how participants made sense of their ability to engage with healthy diet and exercise behavior. Three subthemes were identified, as described below.

##### Subtheme 2.1: Having a Routine and Being Organized

Having structure or a pre-existing routine supported the maintenance of healthy diet and exercise behaviors of participants, as explained by Craig:

*It’s developing routine, routine is very important. So, sort of, wake up in the morning, do the juice, do the meditate, then exercise, then breakfast. It’s in that slot.*

Regular shopping, cooking and menu planning enabled the participants to have healthy meals and snacks at hand when out of the house or when not feeling well enough to prepare food. Choosing healthy foods were linked to upbringing and made it easier to maintain those behaviors later in life; for example, as Kevin explained:

*I’ve never really had much fast food, as growing up as a kid, we never had the opportunity to have fast food and I’ve just kept that going through my adult life.*

A pre-existing exercise routine made it easier for the participants to maintain regular exercise after their cancer diagnosis. Access to a structured exercise program or supervision by an exercise physiologist were viewed helpful to start or maintain an exercise routine, particularly to those who did not exercise regularly prior to their diagnosis. On the other hand, not having exercised before the cancer diagnosis made it harder to engage with and maintain exercise behavior for some, as described by Louise:

*I’ve always been an active person and that’s just the way I am. I mean, I just do it; it’s probably more of a habit now too. We just get up and we just do it and we feel the benefits from it.*

This is in contrast to Andrew:

*… when I was feeling lousy you couldn’t get me interested in doing any exercise, I had problems walking from one place to another, so it didn’t even come into my mind to even try and exercise. Now I just do the normal things around the place … I’ve never done any formal exercise.*

##### Subtheme 2.2: Self-Determination versus Dependence

This subtheme describes self-determination compared with vulnerability and the dependence of participants to cope with treatment-related side-effects and their cancer diagnosis. Barriers to regular exercise included a lack of energy and strength, general malaise, a lack of motivation, age-related physical inability, brittle bones, frailty, dizziness, constraints around work hours, environmental factors, shortness of breath, pain and access constraints to training facilities due to COVID-19. Barriers to healthy eating included taste changes, food aversions, loss of appetite, nausea, disinterest in food, early satiety and shortness of breath.

Participants who displayed positive self-talk and were able to motivate themselves and/or had access to a support network as a source of encouragement for debriefing and motivation were able to overcome the barriers. Strategies included seeking out support from their local community, family members and healthcare professionals, and problem-solving for adequate nutrition and exercise despite treatment side-effects, for example:

*Sometimes, depending on my physical and emotional state, cooking is difficult; that’s why sometimes it’s good for me to do the big cook at lunchtime. I guess it’s a function of age and also the treatments I’ve done. I get tired and sometimes lack the motivation. But you know, there’s a part of me too that works with: ‘Oh Craig you’re looking after yourself and that’s good’.*

(Craig)

and

*I’ve got a dog, which helps tremendously [to exercise]. I’ve got a husband who also is a good walker and keen on exercise, so I have a companion who I walk with every day. So that’s really good.*

(Louise)

On the other hand, some participants were not able to action healthier behavior, referring to unsurmountable treatment-related side-effects, lack of self-confidence, low motivation, age-related factors and underlying chronic conditions that contributed to the inability to adhere to healthier behavior, as described by Barbara:

*I really need to start eating a lot healthier, but I’m hoping that’ll come. With all the chemo, you lose a lot of taste in your taste buds so your good, healthy food, tastes $@#!... where all the lovely, sweet biscuits and the chocolates don’t.*

Another participant said that:

*I understand the importance of both of them [diet and exercise], but for me it’s just I have no energy compared to what I was before … It’s an absolute struggle to get up in the morning … and do anything at home … I plan an activity … and it might be a half an hour activity … and I can do literally nothing else for the rest of the day … I’m trying to get back into becoming more active but I’m finding it very difficult in that I have no energy.*

(George)

The ability to complete daily chores and engage in these activities was viewed as a sign of good health, provided a sense of purpose, and helped those not engaged with exercise to be active, as Andrew explained:

*Depends on need. What I need to do. If I need to walk to the letter box. If I need to walk from one end of the hospital to the other. Even if I am out of breath, you know, I need to do it...*

##### Subtheme 2.3: Knowledge and Access to Information

Participants spoke about how their pre-existing knowledge and knowledge acquired from healthcare professionals or self-education informed their diet and exercise behavior, as explained by Barbara:

*After I went to the cancer expo on Saturday, I found that it was really informative around exercise and nutrition … It’s given me a little bit of … a wakeup call, it made me really understand that they are the two things that’s going to get me through this. They are the two things that I have to pick up on a little bit more than what I’m doing now.*

Information obtained from healthcare professionals was valued by participants and supported adherence to healthy eating behavior. Furthermore, concerns about dietary intake such as alcohol could be clarified with healthcare professionals, which provided relief. Not all participants received information on diet and exercise behavior from healthcare professionals, either due to lack of access or choice, which impacted their behavior, as David explained:

*I can only go on what I read or see on television as such.*

#### 3.1.3. Theme 3. Taking Back Control and Having Hope for the Future

Diet and exercise behavior were viewed as something that were within the participants’ control and provided a sense of empowerment when other parts of life seemed out of their control. Exercise and following a healthy diet were associated with a good future, the possibility of remission, the hope of not having to be on long-term medication, setting oneself up for improved health outcomes, coping with current treatment side-effects and preparing for future treatments. Suzanne describes:

*Right from the beginning, I realized that the only thing I was going to be in control of was my physical health and mental health … I’m not in charge of what’s going in as far as drugs go, but I am in control of what food I’m eating. So, I think I’m pretty strong-minded and especially in exercise and eating well, that to me is what I’m in control of.*

Samuel stated that:

*The outcome of eating healthy is that one of these days that, along with the exercise of course, I may be able to get off this medication altogether. I’d like to give it the best shot I can …*

Working towards a goal served as self-motivation for a number of participants, and included immediate goals such as keeping moving, maintaining a normal life and gaining weight, and long-term goals such as wanting to be around for family, to become fitter and building up strength to resume hobbies, as explained by George:

*My long-term goal is to commence that [martial arts] next year, so I’ve given myself the rest of this year to get fitter so that I could undertake that.*

## 4. Discussion

To the knowledge of the authors, this is the first study to explore the perceptions of older adults with hematological cancer on the role of diet and exercise behavior in navigating daily tasks. Three interrelated themes emerged that highlight strong beliefs regarding the importance of diet and exercise on physical and mental wellbeing, the ability of participants to navigate and overcome the barriers to healthy diet and exercise behavior, as well as the potential of diet and exercise behavior to empower participants to take action and maintain a sense of control over their own health.

Drawing on behavior change theory, the perceptions of participants on the role of diet and exercise behavior were closely aligned to the dimensions of the Health Belief Model [45]. The fear of cancer recurrence and the hope of remission or slower disease progression were linked with a positive attitude towards following diet and exercise guidelines. For participants with a positive attitude towards and belief in the importance of diet and exercise behavior, the physical and mental health benefits outweighed the reported barriers to adhering to diet and exercise guidelines. Support from family members and healthcare providers, as well as personal beliefs and lifestyle habits before a cancer diagnosis, seemed to support adherence to diet and exercise guidelines. Self-efficacy seemed to underpin the perceived ability to engage in diet and exercise behavior, with a lack of adequate support, knowledge and low self-confidence reported to hamper the ability to engage with diet and exercise guidelines. Participants identified several enablers to diet and exercise behavior which aligned with behavior change techniques that could be used in future diet and exercise intervention programs for older HCS, namely knowledge, habit formation, goal setting, problem-solving, social support, self-belief and instruction on how to perform a behavior [46].

The majority of participants valued the importance of choosing healthy meals and a regular exercise regime for physical and mental wellbeing, which is in accordance with the literature [24,30,47]. Extending this body of evidence, the current study enquired specifically about the perceptions of participants on the role of diet and exercise on physical function (i.e., their ability to perform daily tasks). Participants displayed a good understanding of the link between exercise and the ability to perform daily tasks, linking this to increased energy levels, strength and improved mental health, which were all viewed as important factors contributing to the ability and intent to engage with daily tasks. Many participants were aware of the importance of choosing healthy foods for general health and energy provision, but lacked insight into its role in maintaining physical function [28,29,48,49] and reducing the risk of frailty [50]. In addition, despite the value placed on healthy eating and exercise behavior, some participants reported a lack of self-efficacy to implement these behaviors, with only three participants reporting to have received diet and/or exercise advice and instruction from a healthcare professional during their cancer journey. The lack of nutrition education and counseling from clinicians to facilitate healthy eating behavior, particularly after induction treatment, has been reported as an unmet need by HCS [34]. Similarly, the need for instruction and motivational counseling by healthcare professionals is recognized as important for supporting cancer survivors to engage in regular structured exercise [24,51,52] and following a high-quality diet [53].

Lee and coworkers [54] found dietary modification was 4.5 times more likely in a cohort of cancer patients receiving curative or palliative treatment who strongly believed in the importance of nutrition as part of their treatment (OR 4.5, 95% CI 1.95–10.40). This highlights the importance of a positive attitude and belief in the role of diet in cancer treatment, which can be positively influenced by healthcare professionals. A qualitative inquiry into the promotion of following a healthy diet by members of cancer care teams in the United States found that care team members appreciated the importance of discussing dietary change with survivors, but either failed to engage in the conversation or only briefly touched on the subject [55]. Similarly, findings from a global American Society of Clinical Oncology survey on oncology providers’ (*n* = 971) practice patterns and perceptions regarding lifestyle factors showed that the majority viewed it as important to address diet and exercise behavior during and after active treatment [56]. Both of these studies, however, reported system-related barriers to the delivery of health promotion education and interventions. Nevertheless, opportunities exist for improved delivery of health promotion education and support during the cancer journey. For example, Loeliger and coworkers [57] developed a person-centered care pathway using co-design principles to improve the provision of evidence-based nutritional care throughout the nutrition continuum. These types of innovations require systemic change to take full advantage of their potential to ultimately improve the overall health and physical wellbeing of cancer survivors [55,56,57,58]. In summary, improved access to healthcare professionals to facilitate and support self-management of healthy lifestyle behaviors through education and counseling are warranted for older HCS [59]. This is particularly important in those receiving ongoing maintenance treatment because of the increased risk of frailty, malnutrition and impaired physical function during their cancer journey [49,60].

Several participants in the current study associated following a healthy diet and regular exercise with improved health outcomes and the possibility of remission. In addition, having autonomy over diet and exercise behavior provided a sense of empowerment, as it was an aspect of their cancer journey they felt they had control over. Similarly, other cancer survivors reported that health behavior afforded the opportunity for a sense of control [61] and that it was important to reduce the risk of recurrence or progression [61,62]. Interestingly, Leclair and coworkers [63] found greater physical activity in cancer survivors, as well as improved eating behavior through increased fruit and vegetable intake, was associated with higher self-efficacy, which, in turn, was associated with a lower fear of recurrence. These findings highlight the importance of increasing cancer survivors’ sense of control over their own health through the interplay of increased self-efficacy and adherence to health behaviors, thereby increasing hope for future health.

Similar to others [20,23,31,32], a number of barriers and enablers to eating healthy food and regular exercise were identified in the current study. Unique barriers identified in the current study included age-related physical ability and frailty, which warrants consideration in future diet and exercise intervention programs for older cancer survivors. There was, however, a strong focus on overcoming barriers by most participants, which indicated dietary and exercise resilience. Having a structure and being organized supported adherence to both healthy diet and exercise guidelines. Healthy lifestyle behaviors seemed to be easier with pre-existing healthy habits, and HCS in this study spoke about how their childhood eating habits informed healthy choices and their pre-existing exercise routines supported ongoing physical activity. Habituation combined with self-determination, motivation, personal goals and social support were important factors for engaging with healthy lifestyle behaviors. These factors are consistent with the literature on sustaining healthy food choices and physical activity [64,65]. Community connectedness is a unique enabler to healthy lifestyle behavior in healthy community-dwelling older adults [66], and was also apparent in this group of older cancer survivors. Further research is warranted to explore the role of community connection as an opportunity for improved diet and exercise behaviors in older cancer survivors.

Limitations to this study include respondent bias, which may have occurred with the participants opting to partake in the study having a keen interest and strong views on diet and exercise behavior. This study had a large representation of men; therefore, the results and the perceptions relating to diet and exercise behavior may be different with the inclusion of more women in a similar population group. Nevertheless, the proportion of male participants in our sample is similar to prevalence estimates of hematological malignancies, which are higher in males than females. The small sample size prevents generalization of the findings to older HCS but provides valuable insights into the factors affecting diet and exercise behavior that warrant further investigation. One participant was receiving active treatment while all other participants were receiving maintenance treatment, there was a large variation in time since diagnosis between participants, and time since a stem cell transplant was not recorded, which may have influenced the findings. Further research is required to explore the perceptions of the role of diet and exercise behavior in maintaining physical function in a wider HCS population.

## 5. Conclusions

In summary, participants valued the importance of a healthy diet and regular exercise to engaging in daily tasks and supporting physical and mental wellbeing. Several factors contributed to this group of older HCSs’ ability to engage with diet and exercise behavior, of which knowledge, social support, self-belief, habit formation, goal setting, instruction and problem-solving skills supported adherence to diet and exercise guidelines. The study identified similar barriers and enablers to diet and exercise behaviors to those of solid tumor cancer survivors. Furthermore, the barriers and enablers seemed to be independent of age, with the exception of age-related physical decline, which was identified as a barrier, and the potential for community connection as an enabler for healthy diet and exercise behavior. There was a need for improved access to nutrition and exercise education and/or support in this group of older HCS. Finally, regular screening for malnutrition and frailty in older HCS receiving long-term antineoplastic treatment is recommended.

## Figures and Tables

**Table 1:** Demographic information, frailty and malnutrition risk of participants.

Variable	Total Group (*n* = 9)
Age	67 ± 2
Reported height (cm)	172 ± 2.9
Reported weight (kg)	75.6 ± 5.2
Body mass index (kg/m^2^)	25.2 ± 1.0
PRISMA-7 score	2.6 ± 0.4
Malnutrition screening score	0 (0; 2)
Time since diagnosis (months)	12 (10.5; 20)
	***n***
**Marital status**	
Married/cohabitating	8
Single/widowed	1
**Employment status**	
Employed	2
Unemployed	1
Retired	6
**Cancer type**	
Multiple myeloma	3
Chronic myeloid leukemia	1
Myelodysplastic syndrome	1
Non-Hodgkin lymphoma	1
Refractory Hodgkin lymphoma	2
Relapse follicular lymphoma	1
**Treatment stage**	
Induction	1
Consolidation	1
Maintenance	4
Second-line	3
**History of stem cell transplant**	
Yes	4
No	5

Note. Parametric data are reported as the mean ± standard deviation, non-parametric data are reported as the median (IQR). Categorical data are reported as the frequency.

**Table 2:** Characteristics of participants from semi-structured interviews.

Pseudonym Gender	Age	Time Since Diagnosis	Cancer Diagnosis	Physical Activity Level	Frailty Risk	PF ^1^	Malnutrition Risk
George, male	61	12 months	Multiple myeloma	Sufficiently active	At risk	20	Yes
Samuel, male	67	9 months	Chronic myeloid leukemia	Moderately active	At risk	35	No
Kevin, male	67	5 months	Non-Hodgkin lymphoma	Sufficiently active	Not at risk	85	No
Andrew, male	69	13 months	Hodgkin lymphoma	Insufficiently active	At risk	20	No
David, male	70	12 months	Multiple myeloma	Insufficiently active	At risk	55	Yes
Craig, male	71	24 months	Refractory follicular lymphoma	Sufficiently active	At risk	40	No
Suzanne, female	72	6 years	Multiple myeloma	Sufficiently active	Not at risk	40	No
Louise, female	73	16 months	Myelodysplastic syndrome	Sufficiently active	Not at risk	95	No
Barbara, female	76	12 months	Hodgkin lymphoma	Moderately active	Not at risk	65	Yes

^1^ Physical functioning subscale score of SF-36 [39] scores out of 100; a higher score indicates better function.

## Data Availability

Not applicable.
